# The endothelin receptor antagonist macitentan ameliorates endothelin-mediated vasoconstriction and promotes the survival of retinal ganglion cells in rats

**DOI:** 10.3389/fopht.2023.1185755

**Published:** 2023-06-16

**Authors:** Bindu Kodati, Wei Zhang, Shaoqing He, Jennifer H. Pham, Kallen J. Beall, Zoe E. Swanger, Vignesh R. Krishnamoorthy, Payton E. Harris, Trent Hall, Ashley V. Tran, Renuka M. Chaphalkar, Sai H. Chavala, Dorota L. Stankowska, Raghu R. Krishnamoorthy

**Affiliations:** ^1^ Department of Pharmacology and Neuroscience, University of North Texas Health Science Center, Fort Worth, TX, United States; ^2^ North Texas Eye Research Institute, University of North Texas Health Science Center, Fort Worth, TX, United States; ^3^ Department of Pathology, Children’s Health at, Dallas, Dallas, TX, United States; ^4^ Department of General Surgery, Honor Health, Phoenix, AZ, United States; ^5^ Texas College of Osteopathic Medicine, University of North Texas Health Science Center, Fort Worth, TX, United States; ^6^ Department of Cell and Molecular Pharmacology, Loyola University, Maywood, IL, United States; ^7^ Department of Graduate Medical Education, Medical City, Fort Worth, TX, United States; ^8^ Williams College, Williamstown, MA, United States; ^9^ School of Natural Sciences and Mathematics, The University of Texas at Dallas, Richardson, TX, United States; ^10^ Department of Ophthalmology, School of Medicine, University of California, San Francisco, CA, United States; ^11^ Department of Surgery, Burnett School of Medicine at Texas Christian University (TCU), Fort Worth, TX, United States

**Keywords:** glaucoma, endothelins, vasoconstriction, macitentan, neuroprotection

## Abstract

Glaucoma is a chronic and progressive eye disease, commonly associated with elevated intraocular pressure (IOP) and characterized by optic nerve degeneration, cupping of the optic disc, and loss of retinal ganglion cells (RGCs). The pathological changes in glaucoma are triggered by multiple mechanisms and both mechanical effects and vascular factors are thought to contribute to the etiology of glaucoma. Various studies have shown that endothelin-1 (ET-1), a vasoactive peptide, acting through its G protein coupled receptors, ET_A_ and ET_B,_ plays a pathophysiologic role in glaucoma. However, the mechanisms by which ET-1 contribute to neurodegeneration remain to be completely understood. Our laboratory and others demonstrated that macitentan (MAC), a pan endothelin receptor antagonist, has neuroprotective effects in rodent models of IOP elevation. The current study aimed to determine if oral administration of a dual endothelin antagonist, macitentan, could promote neuroprotection in an acute model of intravitreal administration of ET-1. We demonstrate that vasoconstriction following the intravitreal administration of ET-1 was attenuated by dietary administration of the ET_A_/ET_B_ dual receptor antagonist, macitentan (5 mg/kg body weight) in retired breeder Brown Norway rats. ET-1 intravitreal injection produced a 40% loss of RGCs, which was significantly lower in macitentan-treated rats. We also evaluated the expression levels of glial fibrillary acidic protein (GFAP) at 24 h and 7 days post intravitreal administration of ET-1 in Brown Norway rats as well as following ET-1 treatment in cultured human optic nerve head astrocytes. We observed that at the 24 h time point the expression levels of GFAP was upregulated (indicative of glial activation) following intravitreal ET-1 administration in both retina and optic nerve head regions. However, following macitentan administration for 7 days after intravitreal ET-1 administration, we observed an upregulation of GFAP expression, compared to untreated rats injected intravitreally with ET-1 alone. Macitentan treatment in ET-1 administered rats showed protection of RGC somas but was not able to preserve axonal integrity and functionality. The endothelin receptor antagonist, macitentan, has neuroprotective effects in the retinas of Brown Norway rats acting through different mechanisms, including enhancement of RGC survival and reduction of ET-1 mediated vasoconstriction.

## 1 Introduction

**Figure 1:**
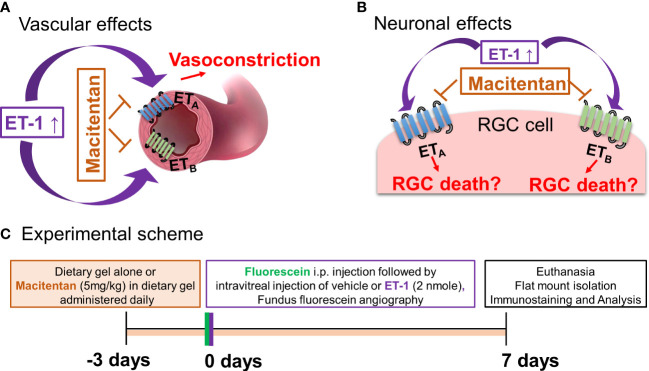
Hypothetical mechanisms of action of endothelin 1 (ET-1). **(A)** Vascular effects of ET-1. **(B)** Effects of ET-1 on the retinal ganglion cells. **(C)** Experimental scheme.

Endothelins are a family of potent 21-amino-acid vasoactive peptides which play important roles in normal physiology by maintaining vascular tone in various organ systems (1, 2) **Figure 1A**. In humans, there are three isoforms of endothelins encoded by separate genes: endothelin-1 (ET-1), endothelin-2 (ET-2), and endothelin-3 (ET-3), which binds to G-protein coupled receptors, ET_A_ and ET_B,_ to produce diverse effects in multiple cell types (3). Several studies have shown that endothelins contribute to the pathophysiology of glaucoma (4–7). ET-1 levels are elevated in the aqueous humor of primary open-angle glaucoma (POAG) patients (8, 9) and also in animal models of glaucoma (5, 6, 10). In particular, endothelin B (ET_B_) receptors have been shown to play a key role in neurodegeneration in animal models of glaucoma (6, 11, 12), **Figure 1B**. Our laboratory also demonstrated the upregulation of the ET_A_ receptor in a rat model of ocular hypertension (13). While the role of the ET_A_ receptor in neurodegeneration is not completely understood, studies have shown that blocking both endothelin receptors (ET_A_ and ET_B_) provides neuroprotection in an inheritable mouse model of glaucoma (14).

Apart from intraocular pressure (IOP) elevation, vascular dysregulation is also thought to contribute to the glaucoma pathophysiology (15–17). ET−1 promotes vasoconstriction by its actions on both endothelin receptors ET_A_ and ET_B_. Hence, one potential mechanism responsible for ET-1’s actions could involve a vascular component, manifested as decreased blood flow to the retina and optic nerve head. In our recent study using a chronic model of IOP elevation, we demonstrated that oral administration of the dual endothelin receptor antagonist, macitentan (5 mg/kg body weight), had neuroprotective effects on RGCs and their axons (18). In glaucoma, there are IOP-dependent as well as IOP-independent factors that contribute to the neurodegeneration of RGCs and their axons (19–22). Although there are models of acute angle closure glaucoma to study the mechanisms of ischemia-reperfusion injury at the optic nerve head and retina, studying the vascular changes mediated by ET-1 will provide additional insight into vascular mechanisms contributing to neurodegeneration in glaucoma. Since ET-1 is known to be a potent vasoconstrictor, we wanted to determine if a dual endothelin antagonist macitentan could attenuate retinal vasoconstriction and promote RGC neuroprotection following an intravitreal administration of an acute dose of ET-1 (2 nmole/eye).

Neuroinflammation is one of the contributors to neurodegeneration in glaucoma, which is initiated through the activation of various glial cells, including astrocytes and microglia in the retina and optic nerve head. Endothelins have been shown to promote astrocyte proliferation and reactive gliosis, which could result in glial scarring and axon loss (23–25). Depending upon the glaucomatous insult and its duration, glial activation may promote either neuroprotective or neurodegenerative effects in various models of glaucoma (26). We also studied the effect of dietary administration of the endothelin receptor antagonist macitentan on the expression of some genes indicative of neuroinflammation by assessing their expression levels in the retina.

## 2 Methods

### 2.1 Animals

Animal studies were performed in accordance with the Association for Research in Vision and Ophthalmology (ARVO) resolution for the Use of Animals in Ophthalmic and Vision Research and approved by the University of North Texas Health Science Center (UNTHSC) Institutional Animal Care and Use Committee (IACUC). Retired breeder male and female Brown Norway rats (8- to 11-months-old) were obtained from Charles River (Wilmington, MA).

### 2.2 Macitentan treatment

Macitentan was a kind gift from Actelion Pharmaceuticals US, Inc. (CA, USA). Macitentan treatment (5 mg/kg body weight/day) was initiated three days prior to the intravitreal injections of ET-1 or vehicle and was continued for an additional 7 days post-intravitreal injection (for a total of 10 days) (**Figure 1C** **)**. To ensure even consumption, macitentan was administered orally by mixing the drug into DietGel^®^ Recovery (Clear H_2_0, Westbrook, ME). Untreated rats were fed with DietGel^®^ alone. Rats were monitored to ensure complete consumption of the medication.

### 2.3 Intravitreal injections of ET-1 or vehicle

Endothelin-1 (Bachem, Torrance, CA, USA) was dissolved either in 0.25% acetic acid ((adjusted to pH 7.0 with sodium hydroxide) or in water to a final concentration of 500 µM. Intravitreal injections were performed using a Hamilton syringe with a 32-gauge needle. The rats were either anesthetized by intraperitoneally injecting an anesthetic cocktail of ketamine (55 mg/kg)/xylazine (5.5 mg/kg)/acepromazine (1.1 mg/kg) or by using isoflurane. A single drop of 0.5% proparacaine hydrochloride (Alcon Laboratories, Inc., Fort Worth, TX, USA) and 1% tropicamide were applied to both eyes. Intravitreal injection of 4 µl of 500 µM ET-1 (2 nmole) or 4 µl or vehicle was carried out in one eye of Brown Norway rats (with continuous observation of the needle in the center of the vitreous cavity to avoid lens injury). The injections were performed through the sclera, approximately 1 mm behind the limbus, and solutions were slowly (~30 s) injected into the vitreous chamber of the eye. To prevent the injected solution from escaping the eye, the needle tip was held on the injected site in the eye for 30 seconds and then gradually withdrawn. A triple antibiotic (neomycin/polymyxin B/bacitracin) was topically applied at the site of injection to prevent infections and allow healing to occur.

### 2.4 IOP measurements

To assess the changes in intraocular pressure (IOP) following intravitreal administration of either ET-1 or vehicle, IOP measurements were carried out on conscious rats using a TonoLab tonometer (iCare, Finland). Rats were handheld gently, but firmly, while IOP measurements were performed at various time points (0 min, 30 min, 2 h, 4 h, 24 h, and 7 days). For each eye, ten IOP readings were recorded and averaged to yield each IOP value, and the IOP exposure was computed in mmHg-days.

### 2.5 Fundus photography and fundus fluorescein angiography

In another set of experiments, Brown Norway rats were anesthetized with a cocktail of ketamine (55 mg/kg)/xylazine (5.5 mg/kg)/acepromazine (1.1 mg/kg), and pupils were dilated using eye drops comprising of a mixture of 0.5% tropicamide and 0.5% phenylephrine hydrochloride. The cornea surface was anesthetized with 0.5% proparacaine hydrochloride and was kept moist with GenTeal Tears lubricant eye gel (Alcon Laboratories Inc., Fort Worth, TX). AK-FLUO (10% sodium fluorescein, Akorn Inc., Lake Forest, IL) was injected intraperitoneally at 1.5 µl/g of body weight. ET-1 was intravitreally injected 3 minutes following administration of fluorescein, and imaging of the retina was carried out. Micron IV retinal imaging microscope (Phoenix Research Laboratories, Pleasanton, California) was used to capture the fundus and fluorescein angiography images. Serial images were captured at different time points, including 5, 10, 15, 20, 25- and 30 minutes following ET-1 injection. The body temperature was maintained at 37°C using a heating pad. Following recovery from anesthesia, animals were provided food and water ad libitum.

### 2.6 Vessel diameter analysis

The rats were either untreated or treated with macitentan and imaged in the Micron IV retinal imaging microscope both prior to ET-1 injection (0 min time point) as well as 10 minutes following the ET-1 injection (10 min time point). Photographs were imported to FIJI software and analyzed through the Vessel Analysis plugin. The boundary of the analyzed region was set using the instrument parameters and the diameter measurement option was chosen. The diameters of the analyzed picture were set as 3.2 mm (800 pixels). Each detectable vein was then manually marked. The Vessel Analysis software allowed for a selection of the area to be drawn around the portion of the vein closer to the optic nerve, which was considered E1 (approximately one-third of the distance between the optic nerve head and periphery of the retina), and the portion of the vein farther away from the optic nerve, which was considered E2 (approximately two-thirds of the distance between the optic nerve head and periphery of the retina). This process was repeated in the same manner for all of the images of the experimental eyes.

### 2.7 Vessel density analysis

The vascular length density and the vascular density were measured using the FIJI Vessel Analysis plugin and an area of the image was selected to focus on the veins without incorporating the blank areas. The region of interest tool was used and a circle with the dimensions of 400×400 pixels was used on all of the images generated from the rat eyes. The vascular length and vascular density were then given and recorded for the veins in the image and calculated as a percentage of the area.

### 2.8 Pattern electroretinography

Rats were anesthetized by intraperitoneal injection (100 μL/100 g body wt) of a ketamine (VEDCO)/xylazine (VEDCO)/acepromazine (Lloyd Laboratories) cocktail with final concentrations of 55.6 mg/mL/5.6 mg/mL/11.1 mg/mL, respectively. Pattern ERG analysis was carried out using the Jörvec instrument (Intelligent Hearing Systems, Miami, FL) (18). Briefly, rats were placed onto a heated platform adapted for rats, which allowed unobstructed views of the visual stimulus monitors. Rats were maintained at 37°C for the duration of the procedure. Reference and ground electrodes were placed subcutaneously in the scalp and base of the tail, respectively. GenTeal eye lubricant/artificial tears (Alcon labs) were applied to both eyes to prevent drying and corneal electrodes were positioned at the lower fornix in contact with the eye globe. LED monitors were used to display contrast-reversing horizontal bars at a spatial frequency of 0.095 cycles/degree and a luminance of 500cd/m^2^. Pattern ERG waveforms for each run consisted of 372 sweeps which were then averaged, processed, and analyzed further to determine PERG amplitude and latency using the PERG software (Jorvec).

### 2.9 Retinal flat mount immunostaining

Following the treatments, animals were euthanized and their eyes were enucleated. The eye cups were fixed overnight at 4°C in 4% paraformaldehyde (PFA) and retinal flat mounts were prepared. To prevent non-specific binding with the secondary antibody, blocking was carried out overnight at 4°C using 5% normal donkey serum and 5% BSA in PBS. Retinal flat mounts were incubated in primary antibody solution, goat anti-Brn3a (1:200; Santa Cruz) for 72 hours at 4°C. Subsequently, secondary antibody incubation was carried out using a 1:1000 dilution of Alexa 647 conjugated donkey anti-goat antibody (Life Technologies, Carlsbad, CA) overnight at 4°C. The retinal flat mounts were mounted on slides using Prolong Gold anti-fade (Life Technologies). All images were taken with 4x magnification in a Cytation 5 cell imaging multimode reader microscope (BioTek, Winooski, VT).

### 2.10 Semi-automatic retinal ganglion cell counting

The fluorescence images of immunostained retinal flat mounts were uploaded to ImageJ, a free photo editor designed for biology research (Rasband, 1997-2018). The images were then converted to 8-bit greyscale to reduce background noise and processed with the automatic nuclei counter plugin “ICTN,” which automatically counts high contrast points within the image. To maintain consistency in cell counts, the ICTN settings were set to detect cells of a specific width, distance apart, and contrast threshold (8, 6, and 1.5, respectively). The cells not detected by the ICTN program were then counted manually by a masked observer and summed as total RGC counts.

### 2.11 Assessment of axonal integrity in optic nerve sections

Axonal degeneration was examined using paraphenylenediamine (PPD) staining, which stains the myelin around the axons. Briefly, following various treatments, rats were humanely euthanized by intraperitoneal administration of Fatal-Plus (pentobarbital: 120 mg/kg body weight), and their eyes were enucleated, after which the optic nerves were excised posterior to the globe. The optic nerves were then immediately fixed in 2% paraformaldehyde, 2.5% glutaraldehyde in 0.1 M sodium cacodylate buffer and processed further. Optic nerve cross-sections were obtained using an ultramicrotome and stained with 1% PPD. Images of PPD stained sections were taken in a Zeiss LSM 510 META confocal microscope using an oil immersion magnification × 100. Images were taken at a few points in the center, as well as the peripheral region of each quadrant of every optic nerve section. The analysis was performed using Image J software for the axon counts (18). The axon counts were compared between the different treatment groups to assess neurodegenerative/neuroprotective effects.

### 2.12 Immunohistochemistry

After various treatments, animals were humanely euthanized by intraperitoneal administration of Fatal-Plus (pentobarbital: 120 mg/kg body weight), following which their eyes were enucleated and immediately fixed in 4% paraformaldehyde (PFA) by making a small incision at the limbal region and placed on a shaker at room temperature for 30 minutes. After the incubation, the posterior part of the globe was completely separated from the anterior segment and lens. Incubation in 4% PFA was continued at room temperature on a shaker for about 3 hours. The fixed retinas were subsequently washed, immersed overnight in 70% ethanol, and thereafter embedded in paraffin and sectioned. Sagittal retinal sections through the optic nerve head were obtained, deparaffinized using xylene, and rehydrated with a graded descending series of ethanol concentrations (100%, 95%, 90%, 80%, and 50% ethanol) and finally with PBS. Permeabilization with 0.1% sodium citrate and 0.1% Triton X-100 was carried out for eight minutes to facilitate the subsequent entry of antibodies. To prevent nonspecific binding of the secondary antibody, the sections were incubated with blocking buffer (5% normal donkey serum and 5% BSA in PBS) for approximately two to three hours. After blocking, the sections were incubated with the respective primary antibodies overnight at 4°C. The primary antibody used for the experiment was GFAP – Chicken (1:500, Abcam – ab4674). After primary antibody incubation, the sections were washed three times with PBS and incubated with appropriate secondary antibodies for one to two hours at room temperature. The secondary antibody used in the experiment was donkey anti-chicken Alexa 647 (1:1000 dilution, A10036; Life Technologies, Carlsbad, CA, USA. To assess the nonspecific binding of secondary antibodies, “blank” sections (in which the primary antibody incubation was omitted) were used after incubation only with the secondary antibody. Retinal sections were mounted with Prolong Gold anti-fade (Life Technologies). Images were taken in the Cytation 5 microscope (BioTek, Winooski, VT) and analyzed using Image J.

### 2.13 Primary optic nerve head astrocytes

Rat primary optic nerve head astrocytes were isolated from adult 8 to 10-week-old female Sprague-Dawley rats and cultured using an astrocyte-selective medium, AM-a medium (ScienCell Research Laboratories, Inc., CA, USA, Catalog #1801) with 2% FBS (27). When cells reached 80% confluence, the culture medium was changed to serum-free astrocyte medium. Pretreatment of the cells was carried out by treatment with 1 μM macitentan or 0.1% DMSO (vehicle) for 30 min. After the pretreatment, the cells were treated with ET-1 at a final concentration of 100 nM for 24 hours.

At the end of the treatment, the cells were harvested using the TRIZOL reagent and the total cellular RNA was extracted following the manufacturer’s instruction. The quantity and quality of total RNA were monitored by Nanodrop 2000 (Fisher Scientific). cDNA was synthesized using BioRad iScript Reverse Transcription Supermix (#1708840), and qPCR was performed to detect the expression of GFAP and fibronectin using BioRad SsoAdvance Universal SYBR Green Supermix (#1725271). Expression of the housekeeping gene cyclophilin served as an internal control. The following primers were used for the qPCR analyses:

Rat GFAP (Sense): 5’-AAATTGCTGGAGGGCGAAGA

Rat GFAP (Anti-sense): 5’-CCGCATCTCCACCGTCTTTA

Rat Fibronectin (Sense): 5’-AAACCGGGAAGAGCAAGAGG

Rat Fibronectin (Anti-sense): 5’-CCTAGGTAGGTCCGTTCCCA

Rat Cyclophilin A (Sense): 5’- CGGAGAGAAATTTGAGGATGA

Rat Cyclophilin A (Anti-sense): 5’- CATCCAGCCACTCAGTCTTG

### 2.14 Statistical analysis

GraphPad prism (GraphPad Software, La Jolla, CA) was used for statistical analyses. The data were expressed as the mean ± standard error (SEM) of three or more independent experiments. Statistical significance for most experiments was evaluated by Two-way ANOVA followed by Tukey’s multiple comparisons test. To determine statistical significance of vessel diameter unpaired t-test at p ≤ 0.02 was used.

## 3 Results

### 3.1 Vasoconstrictive effects of intravitreally injected ET-1 are reduced in rats treated with macitentan in the diet

**Figure 2:**
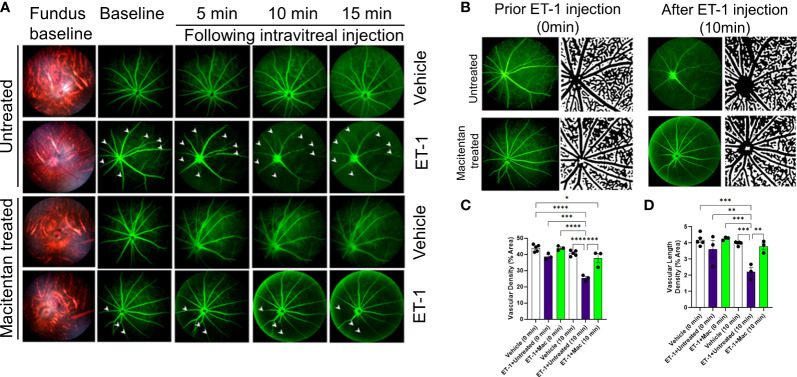
Retinal vasculature fluorescein angiography and vascular density analysis in Brown Norway rats. Rats were either treated or untreated with macitentan (endothelin receptor antagonist) for three days before intravitreal injection of ET-1. **(A)** The retinal vasculature was imaged with the Micron IV microscope prior to ET-1 injection (baseline) and subsequently at 5, 10 and 15 minutes. Arrowheads indicate the blood vessels which had the most prominent vasoconstriction **(B)** The retinal vasculature imaged with the Micron IV microscope prior to ET-1 injection and subsequently at 10 minutes. The processed images are the representative images for the Micron IV. Black color represents blood vessels, imaged prior to and post injection with ET-1 in rats that were either treated or untreated with macitentan.The field of view is equal to 3.6 mm **(C, D)** The vascular density and vascular density length were then measured using FIJI Vessel Analysis plugin and presented as percent of total area Mean ± SEM, n= 3-5 rats per treatment, where (*) = p ≤ 0.05, (**) = p ≤ 0.005, (***) = p ≤ 0.0005 and (****) = p < 0.0001 using two-way ANOVA (Tukey’s multiple comparisons test).

ET-1 could act through vascular mechanisms by its ability to promote vasoconstriction and/or through its direct actions on RGCs to produce cell death (**Figures 1A, B**). **Figure 1C** depicts the experimental scheme that was employed for the current study. To test the ability of the dual ET_A_/ET_B_ antagonist macitentan on ET-1 mediated vascular changes in the eye, we treated male and female Brown Norway rats with either the dietary gel alone or dietary gel containing macitentan (5 mg/kg), starting 3 days prior to the ET-1 intravitreal injection. On the day of the experiment (day “0”), the rats were intraperitoneally injected with sodium fluorescein to visualize retinal vasculature. Three minutes following the intraperitoneal injection of sodium fluorescein, rats were intravitreally injected either with the 4 µl of vehicle or with 4 µl of 500 µM ET-1. Images were captured at various time points, including 0, 5, 10, 15, 20, 25, and 30 min following the intravitreal injections. We observed profound vasoconstriction (particularly in the retinal arteries) in ET-1 injected untreated eyes (**Figure 2A**), which was reflected by a significant decrease in vessel diameter and vascular density, particularly at 10 minutes following ET-1 administration (**Figures 2B-D**).

### 3.2 Effect on vascular density

A detailed analysis was performed on the vasoconstrictive effects at the 10 min time point following ET-1 administration. The mean vascular density at the zero time point for various treatments was as follows. Vehicle-injected rats: 43.9 ± 0.95, untreated ET-1 injected rats: 38.7 ± 1, and macitentan-treated ET-1 injected rats: 43.9 ± 0.8. Between these groups, we did not find any statistical difference at the zero-time point. After 10 minutes following intravitreal administration, the vascular densities for vehicle-treated rats were 40.7 ± 0.76, untreated ET-1 administered rats were 25.4 ± 1, and macitentan-treated ET-1 administered rats were 37.7 ± 2.8. A statistically significant difference (p < 0.05) was observed in ET-1 treated rats at the 10 min time interval when compared to vehicle and macitentan treated ET-1 administered rats at the baseline (0 min time point) (**Figures 2B-D**).

### 3.3 Effect on retinal vessel diameter

**Figure 3:**
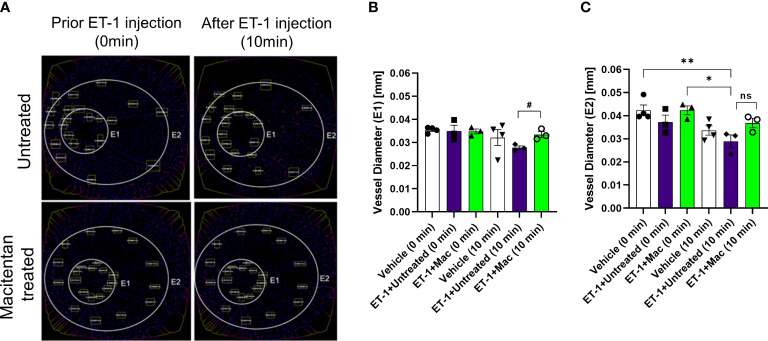
Retinal vessel diameter analysis in Brown Norway rats. Rats were either treated or untreated with macitentan for three days before intravitreal injection of ET-1. **(A)** The vessel diameter was then measured using FIJI Vessel Analysis plugin at two distances from the optic nerve head at eccentricity 1 (E1, **B**) and eccentricity 2 (E2, **C**) and presented as Mean of mm ± SEM. n= 3-4 rats per treatment where (#) = p<0.05, (ns) = no statistical difference for the unpaired t-test. Where (*) = p ≤ 0.05 and (**) = p ≤ 0.005 comparisons using two-way ANOVA (Tukey’s multiple comparisons test).

The vasoconstrictive effects were analyzed at two eccentricities (E1- central region near the optic nerve head in the retina and E2- peripheral region of the retina) by assessing the changes in vessel diameter. At the E1 eccentricity, we found statistically significant changes in the retinal vessel diameter between ET-1 injected untreated and ET-1 injected macitentan treated groups at the 10-minute time interval using unpaired t-test (p ≤ 0.02). At the E2 eccentricity, we observed a statistically significant decrease in the vessel diameter in ET-1 untreated animals at the 10 min time interval (0.029 mm ± 0.003) when compared to vehicle injected (p=0.007) and macitentan-treated ET-1 administered (p=0.02) animals at zero time point using Two-way ANOVA (**Figure 3**).

Overall, based on these observations, rats treated with the ET_A_/ET_B_ antagonist macitentan exhibited significantly lesser vasoconstriction, which was reflected in higher vessel diameter and vascular density, compared to the untreated rats injected with ET-1. The significant constriction was evident as early as 5 min following ET-1 injection and lasted up to 30 minutes. On the other hand, vasoconstriction was delayed (particularly evident at the site of injection), and appreciably lesser vasoconstriction was observed at 10 min in rats administered macitentan in dietary gels compared to that in untreated rats. These findings suggest that blocking both endothelin receptors partially prevents vasoconstriction or appreciably delays its onset. Rats injected with ET-1 and fed with dietary gel alone showed no sign of recovery from vasoconstriction up to 30 min (**Supplementary Figure S1**). There were no changes in vasoconstriction in rats injected with the vehicle at any of the tested time points (for both groups: dietary gel or macitentan administered).

### 3.4 Effect on IOP following intravitreal administration of ET-1

**Figure 4:**
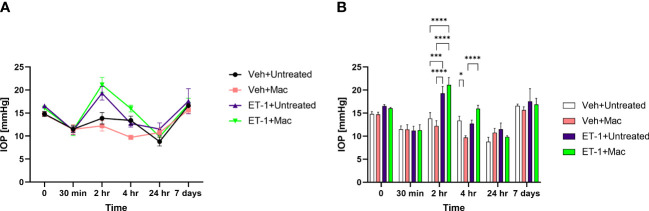
IOP profiles of Brown Norway rats subjected to intravitreal injection either untreated or treated with macitentan. **(A)** IOP measurements in each group are represented by a separate color (Vehicle injected Untreated [black], vehicle injected-macitentan treated [pink], and ET-1 injected untreated [purple], ET-1 injected Macitentan treated [green]). **(B)** IOP values were significantly higher at 2h time points in ET-1 injected animals compared to the vehicle injected animals. The decrease in IOP was observed in ET-1 injected rats at 4 h, 24 h and 7-day time points. Values at each time point represent mean IOP ± SEM; n=3-7 for treatment groups, where (*) = p ≤ 0.05, (***) = p ≤ 0.0005 and (****) = p < 0.0001 using two-way ANOVA (Tukey’s multiple comparisons test).

IOP’s were measured at different time points prior to and post-intravitreal administration of either vehicle or ET-1. In animals following the ET-1 administration (in both untreated as well as macitentan-treated), we observed a significant transient increase in the IOP at the 2 h time point when compared with vehicle-administered animals. The IOPs in the ET-1 injected eyes showed a declining trend at 4 h and the trend continued till the 24 h time point. After 7 days post-treatment, the IOP’s returned to their baseline (**Figure 4**).

### 3.5 ET-1 intravitreal injection produced RGC loss which was significantly attenuated in macitentan-treated rats

**Figure 5:**
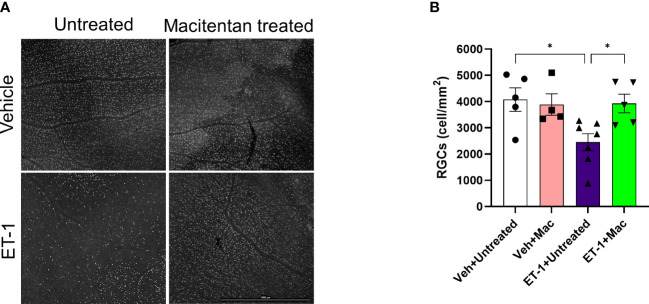
Treatment with macitentan significantly reduces ET-1 mediated RGC loss in Brown Norway rats **(A)** Brown Norway rats were untreated or treated with macitentan (Mac) for 3 days following which they were intravitreally injected with ET-1 or vehicle. Macitentan (or control gel) treatments were continued for additional 7 days. Rats were then sacrificed and retinal flat mounts were isolated. The panel shows representative images of retinal flat mounts immunostained with an antibody to the RGC marker Brn3a. Scale bar represents 1000 µm **(B)** A plot illustrating average number of Brn3a-positive RGCs per field of view (4-8 images per each retina were acquired, n=4-7 rats per group). Scale bar: 1000 μm. Bars represent mean ± SEM. * p<0.05, using two-way ANOVA multiple comparison procedures (Tukey’s Method).

To determine if ET-1 treatment promoted RGC loss and assess the effect of macitentan treatment on RGC survival, macitentan treatment was continued for one week following ET-1 or vehicle injection to one eye of the rats. Following the treatments, rats were euthanized and retinal flat mounts were prepared as described in **Figure 1C**. Labeling of retinal ganglion cells was performed on retinal flat mounts as described previously with minor modifications using an antibody to the RGC marker Brn3a, which labels surviving RGCs (28) (**Figure 5A**). As seen in **Figure 5A**, RGCs have good morphology and are brightly stained using the Brn3a antibody. In contrast, retinas treated with ET-1 show a prominent decrease in Brn3a immunostaining, indicative of both a decrease in Brn3a expression and RGC loss. Dietary macitentan treatment significantly protected against ET-1 mediated RGC loss in rats. **Figure 5B** illustrates the average number of RGCs per retina. Vehicle-injected and untreated have RGC counts of 4076 ± 446 and vehicle-injected macitentan treated has RGC counts of 3886 ± 410 cells per mm^2^. ET-1 injection significantly reduced the number of RGCs by 40% (2454 ± 326) and macitentan treatment significantly (p<0.05) and almost completely reduced the cell loss to 4% (3928 ± 356) compared to vehicle-injected and untreated.

### 3.6 Assessment of RGC functionality following intravitreal injections

In a previous study involving the chronic model of ocular hypertension (Morrison model), we observed a significant increase in RGC survival and an improvement in RGC functionality when the rats were treated with macitentan (5 mg/kg) in the diet. To determine if macitentan will have similar neuroprotective effects following acute ET-1 administration in the eye, we assessed RGC function by performing PERG following intravitreal injection of either ET-1 or its vehicle in rats that were either untreated or treated with macitentan. PERG recordings were taken before the start of the experiment and post-treatment for 7 days.

**Figure 6:**
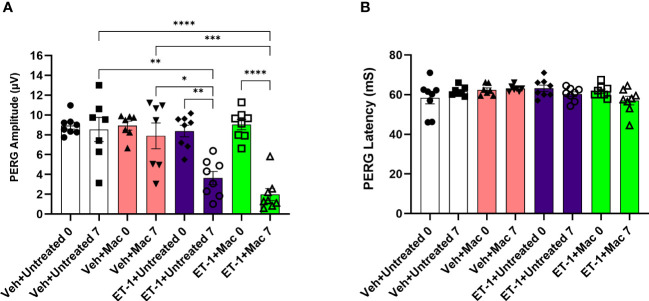
Assessment of retinal ganglion cell (RGC) function in Brown Norway rats following intravitreal injection either with vehicle or ET-1. Pattern electroretinography (ERG) measurements in Vehicle untreated, Vehicle macitentan, ET-1 untreated and ET-1 macitentan-treated retired breeder Brown Norway rats were recorded at prior (Baseline) and post treatment (7 days after intravitreal injection) conditions. **(A)** A significant loss of pattern ERG (PERG) amplitude was observed in animals injected with the ET-1 either treated with gel or macitentan after post treatment compared to their baseline. Comparison between various groups post treatment were only indicated as shown in the analysis **(B)** No difference in the PERG latency between all treatment groups. (*) = p ≤ 0.05, (**) = p ≤ 0.005, (***) = p ≤ 0.0005 and (****) = p < 0.0001 indicates statistical significance using two-way ANOVA (Tukey’s multiple comparisons test), n=7-8 animals per treatment group. Bars represent mean ± SEM.

After the intravitreal injection of the vehicle, in rats that were either treated or untreated with macitentan (5 mg/kg body wt/day), we observed a modest decline (not significant) in the PERG amplitude of the vehicle groups: vehicle and gel treated (at zero-day/baseline: 9.14 ± 2.6 μV and at 7 days post-injection: 7 ± 3.7 μV); vehicle and macitentan treated (at zero-day/baseline: 8.2 ± 2.3 μV and at 7 days post-injection: 7.4 ± 3.5 μV). The ET-1 administered animals showed a significant decline in the amplitude at 7 days compared to day 0, which was not protected by the macitentan treatment: ET-1 and gel treated (at zero-day/baseline 7.9 ± 2.2 μV and at 7 days post-injection 3.3 ± 1.8 μV: p=0.015); ET-1 and macitentan treated (at zero-day/baseline 8 ± 2.6 μV and at 7 days post-injection 2.8 ± 1.7 μV: p=0.002). No significant difference was found in the recorded latency times between untreated and macitentan-treated following the intravitreal injections with either vehicle or ET-1 (**Figure 6**).

### 3.7 Effect of macitentan on axonal integrity following intravitreal administration

**Figure 7:**
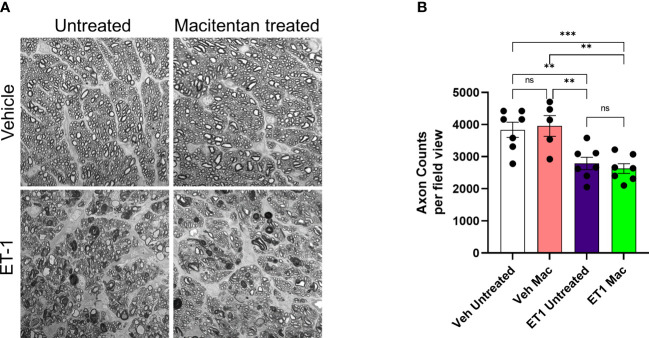
Integrity of optic nerve axons following intravitreal injection either with vehicle and ET-1 and with or without macitentan treatment. Following treatment, rats were euthanized, and optic nerve sections obtained were subjected to PPD staining to assess optic nerve degeneration. Axonal degeneration accompanied by gliosis and glial scar were observed in ET-1 injected untreated rats compared to vehicle injected rat eyes. **(A)** ET-1 injected macitentan-treated rats did not show any protection of their axons, compared to those of ET-1 injected untreated rats. **(B)** The mean counts of healthy axons were significantly reduced in ET-1 injected animals compared to vehicle injected animals. Bars represent mean ± SEM. (**p<0.005, ***p<0.0005) (Two-way ANOVA followed by Tukey’s multiple comparisons test). Scale bar: 20 μm. n=5-7 per treatment group. ns, not significant.

We also assessed the integrity of optic nerve axons in animals treated with or without macitentan following intravitreal administration of either vehicle or ET-1. Seven days post-treatments, rats were euthanized and optic nerve sections stained with PPD were analyzed by confocal microscopy **(** **Figure 7** **)**. When compared with vehicle and untreated animals, ET-1 administered animals showed significant disruption of the optic nerve axonal bundles, intense staining of myelin, and glial scar formation. Based on the axon counts, there was a significant decline in axon counts following intravitreal administration of ET-1, compared to those in vehicle-injected rats (p=0.002). Animals treated with macitentan did not show any protective effect, nor any trend toward the maintenance of axonal integrity when compared between the vehicle and ET-1 administered animals (p=0.003). These findings indicate that in an acute model of ET-1 administration, macitentan could have protective effects on the RGC somas but not on their axons.

### 3.8 ET-1 treatment produced an upregulation of GFAP in rat retinas as well as primary cultures of optic nerve head astrocytes

**Figure 8:**
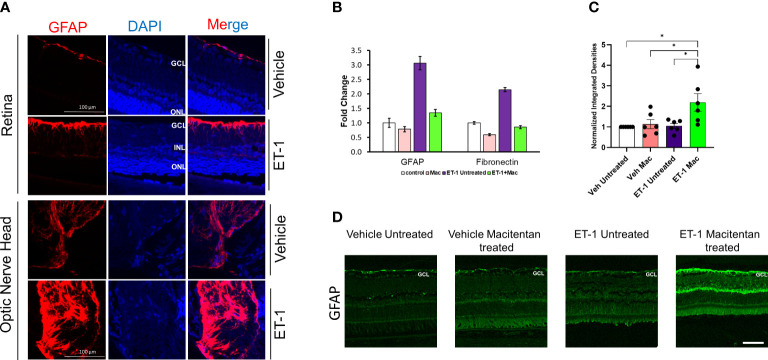
Expression levels of GFAP following intravitreal injections in Brown Norway rats. **(A)** Immunohistochemical analysis of GFAP expression in retinas and optic nerves of Brown Norway rats 24 hours following intravitreal injection of either vehicle or ET-1. Scale bar represents 100 μm **(B)** Quantitative PCR analysis of mRNA expression of GFAP and Fibronectin in rat primary optic nerve head astrocytes isolated from adult Sprague-Dawley rats following 24-hour treatment with either the vehicle, ET-1, macitentan or a combination of ET-1 and macitentan. The graph represents the mean ± SD of one representative experiment. The same experiment was repeated 3 times and similar trend was observed. **(C)** Untreated and macitentan treated rat retinal sections following intravitreal injections with either vehicle or ET-1 post 7 days of the treatment. Images were taken using the Leica DMi8 confocal microscope following immunohistochemical analysis of GFAP. Scale bar represents 75 μm. **(D)** Graph representing the quantification of the normalized integrated densities in the ganglion cell layer. GCL, ganglion cell layer; INL, inner nuclear layer; ONL, outer nuclear layer; DAPI indicated cell nuclei. Bars represent mean ± SEM. (*p<0.05) (Two-way ANOVA followed by Tukey’s multiple comparisons test). n=6 per treatment group.

Astrocytes play a pivotal role in maintaining the structural integrity and ion balance in the cellular environment of retinal neurons, however, following neuronal injury, astrocyte activation, and astrogliosis exacerbate neurodegeneration. To determine if ET-1 could produce activation of astrocytes (since they are in the vicinity of RGCs), we tested retina sections from Brown Norway rats, 24 hours post-intravitreal injection of 2 nmole of ET-1. As seen in **Figure 8A**, increased immunostaining for GFAP (indicative of astrocyte activation) was observed in the nerve fiber layer as well as in the optic nerve head of the rats injected with ET-1, compared to vehicle-injected rats.

Similar to findings in the *in vivo* experiments, cultured primary optic nerve head astrocytes treated with ET-1 (100 nM, 24 hours) showed a similar 3-fold increase in mRNA levels of GFAP, as determined by a q-PCR analysis. Treatment with the ET_A_/ET_B_ dual antagonist, macitentan, completely blocked the ET-1 mediated increase in GFAP mRNA expression. In addition, we also observed a 2-fold increase in mRNA expression of fibronectin, which was prevented by treatment with macitentan. Taken together, ET-1 acting through its receptors has the ability to promote the activation of astrocytes resulting in a pro-fibrotic phenotype, which could have damaging effects on RGCs and their axons (**Figure 8B**).

### 3.9 Effect of macitentan on neuroinflammation following intravitreal administration of ET-1

In order to determine the effect of macitentan on astrocytic activation in the retina, we carried out the immunohistochemistry using the GFAP on retinal sections obtained from the experiment described in **Figure 1C**. Following intravitreal injection of the vehicle and seven days post-treatment with macitentan, we did not observe any difference in the GFAP expression levels when compared between untreated and macitentan treatment after vehicle administration. Similarly, there was no significant difference between untreated ET-1 injected and vehicle-injected (both untreated and macitentan-treated) animals. However, we found a statistically significant difference in expression levels of GFAP with macitentan treated and ET-1 administered animals when compared with all other treatment groups (**Figures 8C, D**).

## 4 Discussion

Members of the endothelin family of vasoactive peptides (including ET-1, ET-2, and ET-3) have been shown to be key players promoting neurodegeneration in glaucoma (4, 29–31). Endothelins could act through both vascular (through its vasoconstrictive effects) and cellular mechanisms (by promoting apoptotic changes in RGCs) to promote degeneration in glaucoma. Vascular mechanisms are difficult to assess in glaucoma patients due to the inter-individual variations in vascular architecture and perfusion of the optic nerve head. Polak et al. (2003) found a decrease in blood flow both in the optic nerve head as well as the choroidal circulation in healthy human subjects following intravenous administration of ET-1, which was blocked by co-administration of an ET_A_ receptor antagonist (32). Several previous studies have shown that continuous perfusion of ET-1 at the optic nerve head could produce a decline in optic nerve head blood in rats, rabbits, and monkeys (7, 33, 34). However, most of these studies did not assess cellular loss of RGCs and additionally did not test the effect of the administration of an endothelin receptor antagonist.

Intravitreal administration of ET-1 into rat eyes could promote loss of RGCs acting through endothelin receptors (11, 23). Using ET_B_-receptor deficient rats, a causative role of the ET_B_ receptor in ET-1mediated loss of RGCs was demonstrated. However, the ability of an endothelin receptor antagonist to protect RGCs was not demonstrated in rats. A recent publication by Marola et al. (2022) reported similar findings of ET-1 mediated vasoconstriction leading to RGC loss (mediated by vascular ET_A_ receptors). However, the authors did not find a direct effect of ET-1 on the viability of RGCs (35). Kiel (2000) found that administration of ET-1 in rabbits decreased choroidal blood flow which was blocked by the non-selective antagonist A-182086, while the ET_A_ antagonist (FR-139317) enhanced the dilation and blocked the constriction (36). These findings raised the possibility of using endothelin receptor antagonists as a neuroprotective agent in glaucoma.

ET-1 has a relatively short half-life (<5 min) in the circulation (37). Hence, the plasma levels of ET-1 may not be reflective of its actual peak concentrations both in the circulation as well as in the aqueous humor. Nevertheless, many publications have demonstrated an increase in ET-1 concentrations in the aqueous humor as well as plasma of glaucoma patients, compared to age-matched control subjects. In our *in vivo* studies, the ET-1 concentrations we used was based on the work done by Stokely et al. (2002) who found a significant decline in fast axonal transport associated with mitochondrial subcomponents following intravitreal administration of 2 mole ET-1 and Lau et al. (2006) who found a significant loss of RGCs following intravitreal administration of 2.5 nmole of ET-1 (38, 39). For studies using mice optic nerve head astrocytes, we had to use 100 nM ET-1. Most studies in culture are unable to detect ET-1 mediated cellular effects at concentrations below 10 to 100 nM ET-1, hence we used a concentration of 100 nM ET-1 in cell culture studies using primary human optic nerve head astrocytes.

Chauhan and colleagues perfused ET-1 through osmotic mini-pumps at the retrobulbar region of the rat eye, for various time durations from 21 to 84 days and found a time-dependent loss of RGCs and damage to their axons (7). Similar to our findings, Lau and colleagues observed 25 to 44% RGC loss (at 1 and 4 weeks respectively) after intravitreal administration of ET-1 (39, 40). This suggests that irrespective of the site of administration, ET-1 could produce neurodegeneration of RGCs and their axons. The precise mechanisms underlying ET-1 mediated neurodegeneration of RGCs are not completely understood. Studies from cardiovascular research have provided some insight into mechanisms by which activation of endothelin receptors could produce cellular damage, some of which may be relevant to neurodegeneration. For instance, perfusion of sarafotoxin (an ET_B_ receptor agonist) was found to greatly elevate superoxide production in the sensory ganglia as well as in glial cells (41). ET-1 acting through its receptors has been shown to decrease glutamate uptake by brain astrocytes (42). ET-1 acting through the ET_B_ receptor produced efflux of glutamate from cultured brain astrocytes, suggestive of its ability to promote excitotoxic effects (43). Previous work from our lab demonstrated that IOP-mediated RGC loss and optic nerve degeneration were significantly attenuated in ET_B_ receptor-deficient rats (12). McGrady showed that ET_A_ receptors are also elevated in the retinas of rats with elevated IOP and have the ability to upregulate ET_B_ expression (13).

In the current study, we have chosen a pan-endothelin receptor antagonist, macitentan, an FDA approved drug to administer orally at 5mg/kg body weight. Compared to other endothelin receptor antagonists such as bosentan and ambrisentan, macitentan has higher affinity, longer receptor occupancy time, and longer half-life (44, 45). Macitentan was effective in lowering mean arterial pressure at a median effective dose (ED_50_) of 1 mg/kg body weight (46). From our recent study, using the chronic model of ocular hypertension, we demonstrated the protective effects of macitentan as this lower dose. Hence, we decided to use a dose of 5 mg/kg body weight. We observed a transient increase in IOP elevation at the 2 h time point following ET-1 intravitreal injection compared to the vehicle injections. The short-term elevation of IOP following intravitreal injection has been observed in rabbits (47) and could be due to ET-1 mediated contraction of the trabecular meshwork (48). A long-term reduction in IOP up to 7 days was reported by MacCumber et al. (1990) in rabbits (49). This could be due to a reduction in aqueous humor formation through its effects on Na+/K+-ATPase (50) and contraction of the ciliary muscle to facilitate aqueous humor drainage. The ET-1 mediated elevation of IOP at the 2 h time point was not attenuated by the oral administration of macitentan (**Figure 4**). The reason for this finding is not clear and could be related to the differential effects of ET-1 on the contraction of ciliary muscle and the trabecular meshwork or due to the acute effects of the bolus administration of ET-1 which is not affected by dietary administration of the endothelin antagonist.

Macitentan treatment was able to enhance RGC survival following ET-1 intravitreal injection. However, its functionality could not be rescued. This points to the drastic effects of acute ET-1 administration and the ensuing RGC injury (similar to that reported by Lau et al., 2006) (39) which cannot be rescued by a dietary administration of the endothelin antagonist. ET-1 treatment compromised axonal integrity (reflected by the demyelination of axons and reactive gliosis in **Figure 7**) and significantly reduced axon counts in the optic nerve. Macitentan treatment was not able to protect against the ET-1 mediated optic nerve axonal degeneration in this acute model. However, in a previous study we were able to protect against the loss of RGCs and their function in a chronic model of ocular hypertension (18). There could be differences in the compartmentalization of gene expression in soma and axons, which could be based on the extent of glaucomatous insult and the animal model of glaucoma being studied. For instance, in a study by Marola et al., 2022, over expression of Bcl-xL protected RGC somas but not the axons in the acute model of optic nerve crush (51). In another study, the over expression of Bcl-xL protected RGC somas and axons in the chronic ocular hypertension model in DBA/2J mice (52). Libby et al. (2005) demonstrated that following optic nerve crush in Bax^-/-^ mice, RGCs are protected from neurodegeneration, however axon loss continues to occur (53). Thus, the somas and axons of RGCs could differ in the susceptibility to glaucomatous insults as well as their survival abilities following neuroprotective treatments.

In the current study, we found that ET-1 intravitreal injection at the 24-hour time point produces activation of rat optic nerve head astrocytes *in vivo*, which was indicated by an increase in GFAP immunostaining in the optic nerve head. Though we observed an upregulation of the GFAP expression levels at the 24 h time point both following ET-1 intravitreal administration as well as following ET-1 treatment in cultured ONH astrocytes, we did not observe an increase in GFAP protein levels 7 days post-intravitreal injection with ET-1 in Brown Norway rats. Similarly, in cultured ONH astrocytes, we found an increase in fibronectin mRNA expression following ET-1 treatment at the 24 h time point and this was appreciably reduced by co-treatment with macitentan. Studies have shown the ability of fibronectin to promote activation of microglia and macrophages into a pro-inflammatory phenotype (54), hence our current findings may point to the ability of macitentan to block these glial changes.

Interestingly, in the ET-1 injected rats, following treatment with macitentan for 7 days, we found a significant increase in GFAP immunostaining in the nerve fiber layer retina compared to that in untreated rats. Mice subjected to a photothrombosis model of stroke, demonstrated a delay/impairment in neurological restoration in GFAP^-/-^Vim^-/-^ mice indicating the importance of GFAP and vimentin in functional recovery and axonal remodeling following stroke (55). Along similar lines, Toops et al. (2012) found that glial cells in retinal explants overexpressing GFAP had more robust neurite outgrowth compared to that of the GFAP^-/-^ mice (56). Additionally, previous studies have shown activated glia are able to promote neurite outgrowth in retinal cultures (57). Perhaps, the acute axonal injury produced by a bolus administration of ET-1 could not be rescued by the upregulation of GFAP at the 7 days timepoint. The involvement of glia in macitentan-mediated neuroprotective effects is not completely clear and will be the subject of future studies.

Howell and colleagues demonstrated that bosentan (dual antagonist of ET_A_ and ET_B_ receptor) treatment promoted neuroprotection of optic nerve axons in the congenital DBA/2J model of glaucoma in mice (14). However, the authors did not assess the effects of bosentan on vascular changes in the retina following endothelin administration. Macitentan has higher potency and efficacy (due to higher receptor occupancy times) as an endothelin receptor antagonist, compared to bosentan (46, 58).). Even though in our present study we have not been assessed tissue levels of macitentan following treatment of animals with macitentan, several studies have shown cytoprotective/neuroprotective effects in various tissues following oral administration of macitentan. For instance, Sen et al. (2012) found the elevation of mRNA expression of extracellular matrix components, namely, fibronectin and collagen α-1(IV) in the retina of diabetic rats, which were significantly attenuated by oral treatment with macitentan (25 mg/kg body wt). Similar damaging effects of endothelin on other organs including the heart, and kidney were also ameliorated by oral administration of macitentan (25 mg/kg body wt) by its ability to prevent increased production of vasoactive and fibrogenic factors following 2- and 4-months of diabetes. In addition, macitentan treatment prevented diabetes-induced VEGF upregulation in these organs (59) and was effective in lowering mean arterial pressure at a median effective dose (ED50) of 1 mg/kg body weight (46). Howell et al. (2014) found appreciable axoprotection in 80% of 10-month-old DBA/2J mice treated with macitentan (30 mg/kg body wt). In addition, the authors used bosentan (100 mg/kg body wt) in C1q knockout DBA/2J mice to show appreciable neuroprotection of optic nerve axons in 80% of 12-month-old animals. However, Howell et al. (2014) did not assess RGC survival, which is reported in this study (60). Based upon all these studies, it is plausible that orally administered macitentan reaches various tissues at pharmacologically effective doses and produces various cellular protective effects through blockade of endothelin receptors.

In summary, the novel aspects of this study include the use of macitentan (5 mg/kg) to generate protective effects including amelioration of retinal vasoconstriction, enhancement of RGC survival, as well as reduction of optic nerve head astrocyte activation in an acute model of ET-1 mediated neurodegeneration. Studies have shown that the use of a similar dose in humans did not produce any major adverse effects. Minor side-effects including headache and back pain during this treatment were also observed in the placebo group (61). Findings from the current study have major implications for the use of orally administered macitentan (which is FDA approved for use in pulmonary hypertension) as a neuroprotective adjunct therapy to IOP-lowering drugs for the treatment of glaucoma. Additionally, our current findings indicate that macitentan has the potential to combat vasoconstrictive effects observed in related clinical conditions, including acute angle closure glaucoma, central retinal artery/vein occlusion, and related ischemic disorders of the retina.

## 5 Conclusions

1. Vasoconstrictive effects following intravitreal ET-1 injection were greatly reduced in rats administered macitentan in the diet prior to the ET-1 administration.
2. ET-1 intravitreal injection produced a 40% loss of RGCs which was significantly reduced in macitentan-treated rats. RGC counts following ET-1 injection and macitentan treatment were similar to that observed in control retinas.
3. Macitentan has some neuroprotective effects in the retinas of Brown Norway rats that possibly occur through different mechanisms, including reduction of ET-1 mediated vasoconstriction, and enhancement of RGC survival. However, RGC functional impairments and axonal injury were not ameliorated in this acute model of ET-1 administration.

## Data availability statement

The original contributions presented in the study are included in the article/**Supplementary materials**, further inquiries can be directed to the corresponding authors.

## Ethics statement

The animal study was reviewed and approved by Institutional Animal Care and Use Committee (IACUC) at the UNT Health Science Center, Fort Worth.

## Author contributions

RK, DS, BK: Conceptualization and Designing experiments, Validation, Writing—Original Draft Preparation, Writing—Review and Editing. WZ, SH, JP, KB, ZS, TH, VK, PH, AT, RC: Assisted in conducting key experiments, Data Analysis. SC: Experimental design. All authors were involved in discussions about the results and their interpretation.

## References

[1] Dhaun N Webb DJ. Endothelins in cardiovascular biology and therapeutics. Nat Rev Cardiol (2019) 16(8):491–502. doi: 10.1038/s41569-019-0176-3 30867577

[2] Yanagisawa M Inoue A Ishikawa T Kasuya Y Kimura S Kumagaye S. Primary structure, synthesis, and biological activity of rat endothelin, an endothelium-derived vasoconstrictor peptide. Proc Natl Acad Sci U S A. (1988) 85(18):6964–7. doi: 10.1073/pnas.85.18.6964 PMC282099 3045827

[3] Inoue A Yanagisawa M Kimura S Kasuya Y Miyauchi T Goto K. The human endothelin family: three structurally and pharmacologically distinct isopeptides predicted by three separate genes. Proc Natl Acad Sci U S A. (1989) 86(8):2863–7. doi: 10.1073/pnas.86.8.2863 PMC287019 2649896

[4] Yorio T Krishnamoorthy R Prasanna G. Endothelin: is it a contributor to glaucoma pathophysiology? J Glaucoma (2002) 11(3):259–70. doi: 10.1097/00061198-200206000-00016 12140405

[5] Kallberg ME Brooks DE Garcia-Sanchez GA Komaromy AM Szabo NJ Tian L. Endothelin 1 levels in the aqueous humor of dogs with glaucoma. J Glaucoma. (2002) 11(2):105–9. doi: 10.1097/00061198-200204000-00005 11912357

[6] Prasanna G Hulet C Desai D Krishnamoorthy RR Narayan S Brun AM. Effect of elevated intraocular pressure on endothelin-1 in a rat model of glaucoma. Pharmacol Res (2005) 51(1):41–50. doi: 10.1016/j.phrs.2004.04.006 15519534

[7] Chauhan BC LeVatte TL Jollimore CA Yu PK Reitsamer HA Kelly ME. Model of endothelin-1-induced chronic optic neuropathy in rat. Invest Ophthalmol Vis Sci (2004) 45(1):144–52. doi: 10.1167/iovs.03-0687 14691166

[8] Noske W Hensen J Wiederholt M. Endothelin-like immunoreactivity in aqueous humor of patients with primary open-angle glaucoma and cataract. Graefes Arch Clin Exp Ophthalmol (1997) 235(9):551–2. doi: 10.1007/BF00947082 9342603

[9] Tezel G Kass MA Kolker AE Becker B Wax MB. Plasma and aqueous humor endothelin levels in primary open-angle glaucoma. J Glaucoma. (1997) 6(2):83–9. doi: 10.1097/00061198-199704000-00003 9098815

[10] Thanos S Naskar R. Correlation between retinal ganglion cell death and chronically developing inherited glaucoma in a new rat mutant. Exp Eye Res (2004) 79(1):119–29. doi: 10.1016/j.exer.2004.02.005 15183107

[11] Krishnamoorthy RR Rao VR Dauphin R Prasanna G Johnson C Yorio T. Role of the ETB receptor in retinal ganglion cell death in glaucoma. Can J Physiol Pharmacol (2008) 86(6):380–93. doi: 10.1139/Y08-040 18516102

[12] Minton AZ Phatak NR Stankowska DL He S Ma HY Mueller BH. Endothelin b receptors contribute to retinal ganglion cell loss in a rat model of glaucoma. PloS One (2012) 7(8):e43199. doi: 10.1371/journal.pone.0043199 22916224 PMC3423444

[13] McGrady NR Minton AZ Stankowska DL He S Jefferies HB Krishnamoorthy RR. Upregulation of the endothelin a (ET(A)) receptor and its association with neurodegeneration in a rodent model of glaucoma. BMC Neurosci (2017) 18(1):27. doi: 10.1186/s12868-017-0346-3 28249604 PMC5333388

[14] Howell GR Macalinao DG Sousa GL Walden M Soto I Kneeland SC. Molecular clustering identifies complement and endothelin induction as early events in a mouse model of glaucoma. J Clin Invest. (2011) 121(4):1429–44. doi: 10.1172/JCI44646 PMC3069778 21383504

[15] Flammer J. The vascular concept of glaucoma. Surv Ophthalmol (1994) 38 Suppl:S3–6. doi: 10.1016/0039-6257(94)90041-8 7940146

[16] Trivli A Koliarakis I Terzidou C Goulielmos GN Siganos CS Spandidos DA. Normal-tension glaucoma: pathogenesis and genetics. Exp Ther Med (2019) 17(1):563–74. doi: 10.3892/etm.2018.7011 PMC6307418 30651837

[17] Flammer J Orgul S Costa VP Orzalesi N Krieglstein GK Serra LM. The impact of ocular blood flow in glaucoma. Prog Retin Eye Res (2002) 21(4):359–93. doi: 10.1016/S1350-9462(02)00008-3 12150988

[18] Kodati B McGrady NR Jefferies HB Stankowska DL Krishnamoorthy RR. Oral administration of a dual ET(A)/ET(B) receptor antagonist promotes neuroprotection in a rodent model of glaucoma. Mol Vis (2022) 28:165–77. PMC9491150 36274816

[19] Ernest PJ Schouten JS Beckers HJ Hendrikse F Prins MH Webers CA. An evidence-based review of prognostic factors for glaucomatous visual field progression. Ophthalmology (2013) 120(3):512–9. doi: 10.1016/j.ophtha.2012.09.005 23211636

[20] Kamal D Hitchings R. Normal tension glaucoma–a practical approach. Br J Ophthalmol (1998) 82(7):835–40. doi: 10.1136/bjo.82.7.835 PMC1722650 9924383

[21] Goldberg I. Relationship between intraocular pressure and preservation of visual field in glaucoma. Surv Ophthalmol (2003) 48 Suppl 1:S3–7. doi: 10.1016/S0039-6257(03)00006-7 12852428

[22] Nakazawa T. Ocular blood flow and influencing factors for glaucoma. Asia Pac J Ophthalmol (Phila). (2016) 5(1):38–44. doi: 10.1097/APO.0000000000000183 26886118

[23] Wang L Fortune B Cull G Dong J Cioffi GA. Endothelin b receptor in human glaucoma and experimentally induced optic nerve damage. Arch Ophthalmol (2006) 124(5):717–24. doi: 10.1001/archopht.124.5.717 16682595

[24] Tezel G Fourth APORICWG. The role of glia, mitochondria, and the immune system in glaucoma. Invest Ophthalmol Vis Sci (2009) 50(3):1001–12. doi: 10.1167/iovs.08-2717 19244206

[25] Prasanna G Krishnamoorthy R Clark AF Wordinger RJ Yorio T. Human optic nerve head astrocytes as a target for endothelin-1. Invest Ophthalmol Vis Sci (2002) 43(8):2704–13. 12147606

[26] Garcia-Bermudez MY Freude KK Mouhammad ZA van Wijngaarden P Martin KK Kolko M. Glial cells in glaucoma: friends, foes, and potential therapeutic targets. Front Neurol (2021) 12:624983. doi: 10.3389/fneur.2021.624983 33796062 PMC8007906

[27] Liu Y Patel GC Mao W Clark AF. Establishment of a conditionally immortalized mouse optic nerve astrocyte line. Exp Eye Res (2018) 176:188–95. doi: 10.1016/j.exer.2018.07.011 PMC6215719 30006274

[28] Stankowska DL Mueller BH 2nd Oku H Ikeda T Dibas A. Neuroprotective effects of inhibitors of acid-sensing ion channels (ASICs) in optic nerve crush model in rodents. Curr Eye Res (2018) 43(1):84–95. doi: 10.1080/02713683.2017.1383442 29111855

[29] Good TJ Kahook MY. The role of endothelin in the pathophysiology of glaucoma. Expert Opin Ther Targets. (2010) 14(6):647–54. doi: 10.1517/14728222.2010.487065 20455789

[30] Rosenthal R Fromm M. Endothelin antagonism as an active principle for glaucoma therapy. Br J Pharmacol (2011) 162(4):806–16. doi: 10.1111/j.1476-5381.2010.01103.x PMC3042192 21054341

[31] Shoshani YZ Harris A Shoja MM Rusia D Siesky B Arieli Y. Endothelin and its suspected role in the pathogenesis and possible treatment of glaucoma. Curr Eye Res (2012) 37(1):1–11. doi: 10.3109/02713683.2011.622849 22029631

[32] Polak K Luksch A Frank B Jandrasits K Polska E Schmetterer L. Regulation of human retinal blood flow by endothelin-1. Exp Eye Res (2003) 76(5):633–40. doi: 10.1016/S0014-4835(02)00312-3 12697427

[33] Cioffi GA Sullivan P. The effect of chronic ischemia on the primate optic nerve. Eur J Ophthalmol (1999) 9 Suppl 1:S34–6. doi: 10.1177/112067219900901S12 10230604

[34] Orgul S Cioffi GA Bacon DR Van Buskirk EM. An endothelin-1-induced model of chronic optic nerve ischemia in rhesus monkeys. J Glaucoma. (1996) 5(2):135–8. 8795746

[35] Marola OJ Howell GR Libby RT. Vascular derived endothelin receptor a controls endothelin-induced retinal ganglion cell death. Cell Death Discovery (2022) 8(1):207. doi: 10.1038/s41420-022-00985-8 35429992 PMC9013356

[36] Kiel JW. Endothelin modulation of choroidal blood flow in the rabbit. Exp Eye Res (2000) 71(6):543–50. doi: 10.1006/exer.2000.0911 11095906

[37] Dupuis J Stewart DJ Cernacek P Gosselin G. Human pulmonary circulation is an important site for both clearance and production of endothelin-1. Circulation (1996) 94(7):1578–84. doi: 10.1161/01.CIR.94.7.1578 8840847

[38] Stokely ME Brady ST Yorio T. Effects of endothelin-1 on components of anterograde axonal transport in optic nerve. Invest Ophthalmol Vis Sci (2002) 43(10):3223–30. 12356828

[39] Lau J Dang M Hockmann K Ball AK. Effects of acute delivery of endothelin-1 on retinal ganglion cell loss in the rat. Exp Eye Res (2006) 82(1):132–45. doi: 10.1016/j.exer.2005.06.002 16045909

[40] Kodati B Stankowska DL Krishnamoorthy VR Krishnamoorthy RR. Involvement of c-jun n-terminal kinase 2 (JNK2) in endothelin-1 (ET-1) mediated neurodegeneration of retinal ganglion cells. Invest Ophthalmol Vis Sci (2021) 62(6):13. doi: 10.1167/iovs.62.6.13 PMC8131991 33978676

[41] Lau YE Galligan JJ Kreulen DL Fink GD. Activation of ETB receptors increases superoxide levels in sympathetic ganglia in vivo. Am J Physiol Regul Integr Comp Physiol (2006) 290(1):R90–5. doi: 10.1152/ajpregu.00505.2005 16179487

[42] Leonova J Thorlin T Aberg ND Eriksson PS Ronnback L Hansson E. Endothelin-1 decreases glutamate uptake in primary cultured rat astrocytes. Am J Physiol Cell Physiol (2001) 281(5):C1495–503. doi: 10.1152/ajpcell.2001.281.5.C1495 11600412

[43] Sasaki Y Takimoto M Oda K Fruh T Takai M Okada T. Endothelin evokes efflux of glutamate in cultures of rat astrocytes. J Neurochem (1997) 68(5):2194–200. doi: 10.1046/j.1471-4159.1997.68052194.x 9109548

[44] Kholdani CA Fares WH Trow TK. Macitentan for the treatment of pulmonary arterial hypertension. Vasc Health Risk Manage (2014) 10:665–73. doi: 10.2147/VHRM.S33904 PMC4251661 25473292

[45] Sidharta PN van Giersbergen PL Dingemanse J. Safety, tolerability, pharmacokinetics, and pharmacodynamics of macitentan, an endothelin receptor antagonist, in an ascending multiple-dose study in healthy subjects. J Clin Pharmacol (2013) 53(11):1131–8. doi: 10.1002/jcph.152 23900878

[46] Iglarz M Binkert C Morrison K Fischli W Gatfield J Treiber A. Pharmacology of macitentan, an orally active tissue-targeting dual endothelin receptor antagonist. J Pharmacol Exp Ther (2008) 327(3):736–45. doi: 10.1124/jpet.108.142976 18780830

[47] Sugiyama K Haque MS Okada K Taniguchi T Kitazawa Y. Intraocular pressure response to intravitreal injection of endothelin-1 and the mediatory role of ETA receptor, ETB receptor, and cyclooxygenase products in rabbits. Curr Eye Res (1995) 14(6):479–86. doi: 10.3109/02713689509003759 7671630

[48] Dismuke WM Liang J Overby DR Stamer WD. Concentration-related effects of nitric oxide and endothelin-1 on human trabecular meshwork cell contractility. Exp Eye Res (2014) 120:28–35. doi: 10.1016/j.exer.2013.12.012 24374036 PMC3943640

[49] MacCumber MW Jampel HD Snyder SH. Ocular effects of the endothelins. abundant peptides in the eye. Arch Ophthalmol (1991) 109(5):705–9. doi: 10.1001/archopht.1991.01080050121041 1755868

[50] Prasanna G Dibas A Hulet C Yorio T. Inhibition of Na(+)/K(+)-atpase by endothelin-1 in human nonpigmented ciliary epithelial cells. J Pharmacol Exp Ther (2001) 296(3):966–71. doi: 10.1038/s41420-022-01111-4 11181930

[51] Marola OJ Yablonski SER Shrager PG Nickells RW Libby RT. BclX(L) (Bcl2l1) gene therapy lessens retinal ganglion cell soma loss but not axonal degeneration after acute axonal injury. Cell Death Discovery (2022) 8(1):331. doi: 10.1038/s41420-022-01111-4 35869049 PMC9307748

[52] Donahue RJ Fehrman RL Gustafson JR Nickells RW. BCLX(L) gene therapy moderates neuropathology in the DBA/2J mouse model of inherited glaucoma. Cell Death Dis (2021) 12(8):781. doi: 10.1038/s41419-021-04068-x 34376637 PMC8355227

[53] Libby RT Li Y Savinova OV Barter J Smith RS Nickells RW. Susceptibility to neurodegeneration in a glaucoma is modified by bax gene dosage. PloS Genet (2005) 1(1):17–26. doi: 10.1371/journal.pgen.0010004 16103918 PMC1183523

[54] Sikkema AH Stoffels JMJ Wang P Basedow FJ Bulsink R Bajramovic JJ. Fibronectin aggregates promote features of a classically and alternatively activated phenotype in macrophages. J Neuroinflammation. (2018) 15(1):218. doi: 10.1186/s12974-018-1238-x 30071854 PMC6091019

[55] Liu Z Li Y Cui Y Roberts C Lu M Wilhelmsson U. Beneficial effects of gfap/vimentin reactive astrocytes for axonal remodeling and motor behavioral recovery in mice after stroke. Glia (2014) 62(12):2022–33. doi: 10.1002/glia.22723 PMC4307923 25043249

[56] Toops KA Hagemann TL Messing A Nickells RW. The effect of glial fibrillary acidic protein expression on neurite outgrowth from retinal explants in a permissive environment. BMC Res Notes. (2012) 5:693. doi: 10.1186/1756-0500-5-693 23259929 PMC3544725

[57] Lorber B Berry M Douglas MR Nakazawa T Logan A. Activated retinal glia promote neurite outgrowth of retinal ganglion cells via apolipoprotein e. J Neurosci Res (2009) 87(12):2645–52. doi: 10.1002/jnr.22095 19382209

[58] Gatfield J Mueller Grandjean C Sasse T Clozel M Nayler O. Slow receptor dissociation kinetics differentiate macitentan from other endothelin receptor antagonists in pulmonary arterial smooth muscle cells. PloS One (2012) 7(10):e47662. doi: 10.1371/journal.pone.0047662 23077657 PMC3471877

[59] Sen S Chen S Feng B Iglarz M Chakrabarti S. Renal, retinal and cardiac changes in type 2 diabetes are attenuated by macitentan, a dual endothelin receptor antagonist. Life Sci (2012) 91(13-14):658–68. doi: 10.1016/j.lfs.2012.03.032 22525377

[60] Howell GR MacNicoll KH Braine CE Soto I Macalinao DG Sousa GL. Combinatorial targeting of early pathways profoundly inhibits neurodegeneration in a mouse model of glaucoma. Neurobiol Dis (2014) 71:44–52. doi: 10.1016/j.nbd.2014.07.016 25132557 PMC4319373

[61] Sidharta PN van Giersbergen PL Halabi A Dingemanse J. Macitentan: entry-into-humans study with a new endothelin receptor antagonist. Eur J Clin Pharmacol (2011) 67(10):977–84. doi: 10.1007/s00228-011-1043-2 PMC3169777 21541781

