# Supplementary figures and images for: The endothelin receptor antagonist macitentan ameliorates endothelin-mediated vasoconstriction and promotes the survival of retinal ganglion cells in rats
Source: Front Ophthalmol (Lausanne). Author manuscript; Available in PMC 2024 Mar 8. (PMC10921982; doi:10.3389/fopht.2023.1185755)

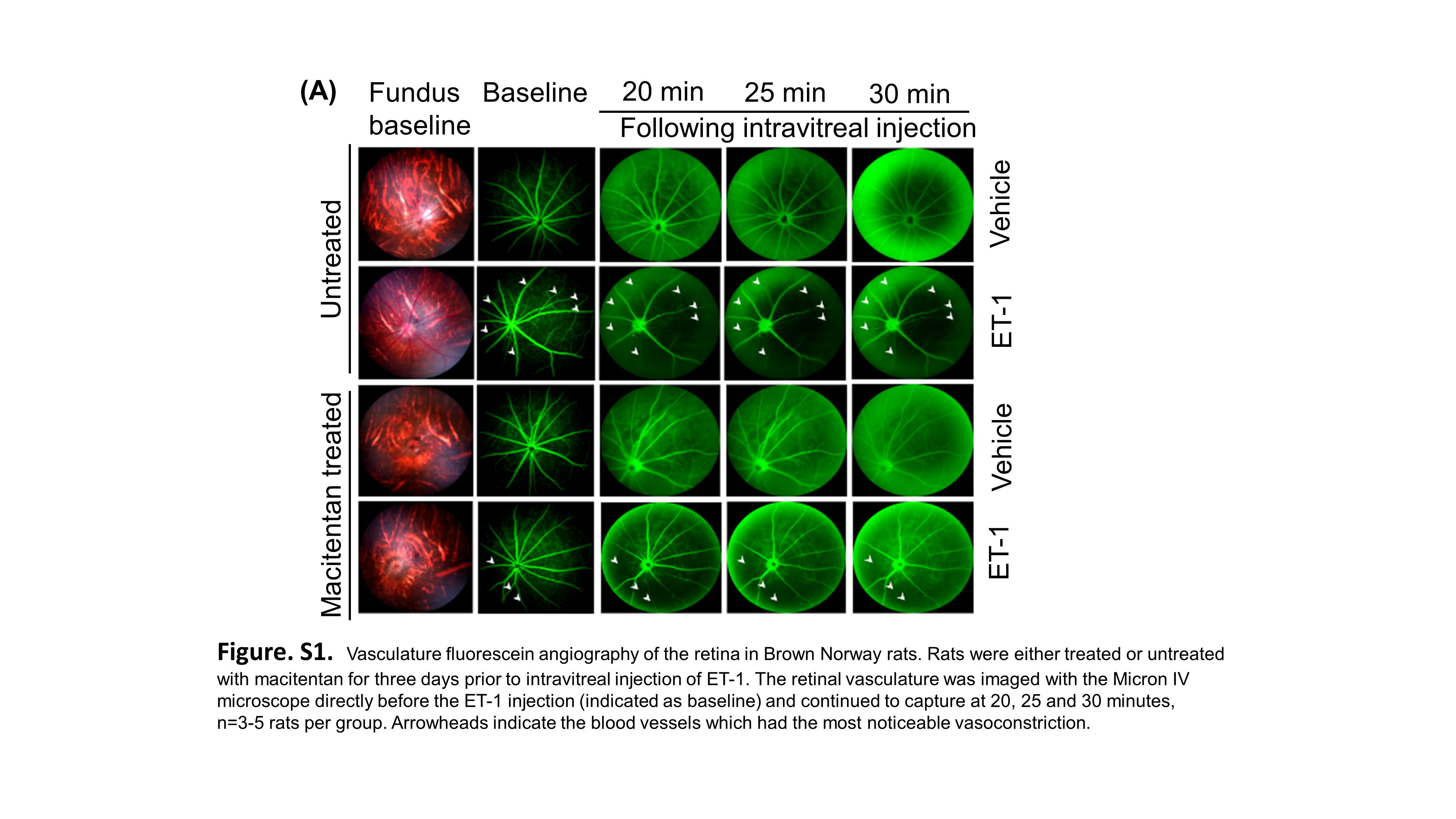

Supplement: Figure S1 [file NIHMS1967644-supplement-Figure_S1.tif]
